# Supplementary material for: Molecular Epidemiology of Xanthomonas euvesicatoria Strains from the Balkan Peninsula Revealed by a New Multiple-Locus Variable-Number Tandem-Repeat Analysis Scheme
Source: Microorganisms. 2021 Mar 5;9(3):536. doi: 10.3390/microorganisms9030536 (PMC8002079; doi:10.3390/microorganisms9030536)
Supplement: Supplementary file 1 [file microorganisms-09-00536-s001.zip › VANCHEVA-Figure_S1.pptx]

## Slide 1
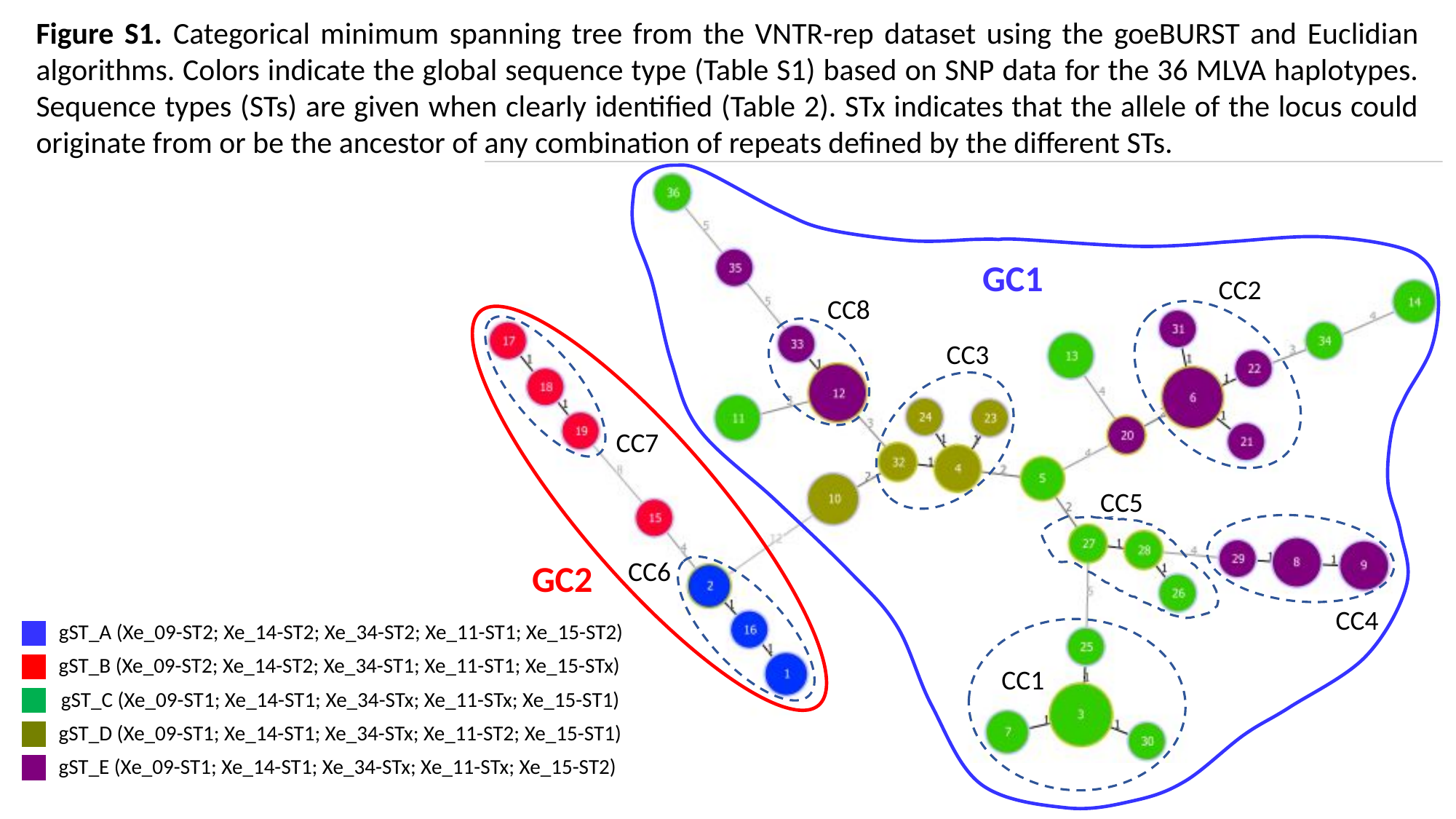

Figure S1. Categorical minimum spanning tree from the VNTR-rep dataset using the goeBURST and Euclidian algorithms. Colors indicate the global sequence type (Table S1) based on SNP data for the 36 MLVA haplotypes. Sequence types (STs) are given when clearly identified (Table 2). STx indicates that the allele of the locus could originate from or be the ancestor of any combination of repeats defined by the different STs.
GC1
CC2
CC8
CC3
CC7
CC5
CC6
GC2
CC4
CC1
gST_A (Xe_09-ST2; Xe_14-ST2; Xe_34-ST2; Xe_11-ST1; Xe_15-ST2)
gST_B (Xe_09-ST2; Xe_14-ST2; Xe_34-ST1; Xe_11-ST1; Xe_15-STx)
gST_C (Xe_09-ST1; Xe_14-ST1; Xe_34-STx; Xe_11-STx; Xe_15-ST1)
gST_D (Xe_09-ST1; Xe_14-ST1; Xe_34-STx; Xe_11-ST2; Xe_15-ST1)
gST_E (Xe_09-ST1; Xe_14-ST1; Xe_34-STx; Xe_11-STx; Xe_15-ST2)
