# Supplementary material for: Molecular Epidemiology of Xanthomonas euvesicatoria Strains from the Balkan Peninsula Revealed by a New Multiple-Locus Variable-Number Tandem-Repeat Analysis Scheme
Source: Microorganisms. 2021 Mar 5;9(3):536. doi: 10.3390/microorganisms9030536 (PMC8002079; doi:10.3390/microorganisms9030536)
Supplement: Supplementary file 1 [file microorganisms-09-00536-s001.zip › VANCHEVA-Figure_S2.pptx]

## Slide 1
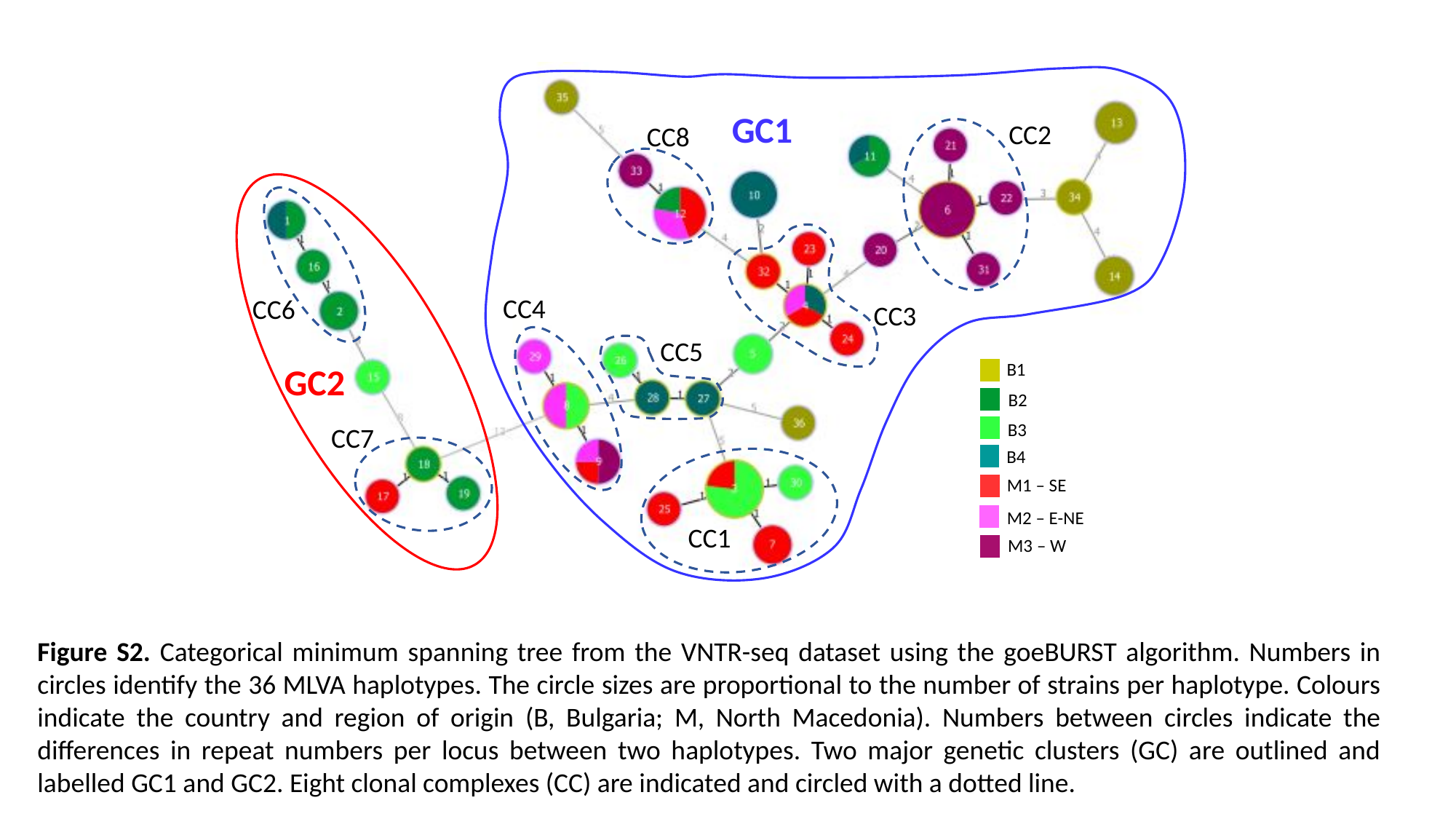

GC1
CC2
CC8
CC4
CC6
CC3
CC5
B1
B2
B3
B4
M1 – SE
M2 – E-NE
M3 – W
GC2
CC7
CC1
Figure S2. Categorical minimum spanning tree from the VNTR-seq dataset using the goeBURST algorithm. Numbers in circles identify the 36 MLVA haplotypes. The circle sizes are proportional to the number of strains per haplotype. Colours indicate the country and region of origin (B, Bulgaria; M, North Macedonia). Numbers between circles indicate the differences in repeat numbers per locus between two haplotypes. Two major genetic clusters (GC) are outlined and labelled GC1 and GC2. Eight clonal complexes (CC) are indicated and circled with a dotted line.
