# Supplementary material for: Molecular Epidemiology of Xanthomonas euvesicatoria Strains from the Balkan Peninsula Revealed by a New Multiple-Locus Variable-Number Tandem-Repeat Analysis Scheme
Source: Microorganisms. 2021 Mar 5;9(3):536. doi: 10.3390/microorganisms9030536 (PMC8002079; doi:10.3390/microorganisms9030536)
Supplement: Supplementary file 1 [file microorganisms-09-00536-s001.zip › VANCHEVA-Figure_S3.pptx]

## Slide 1
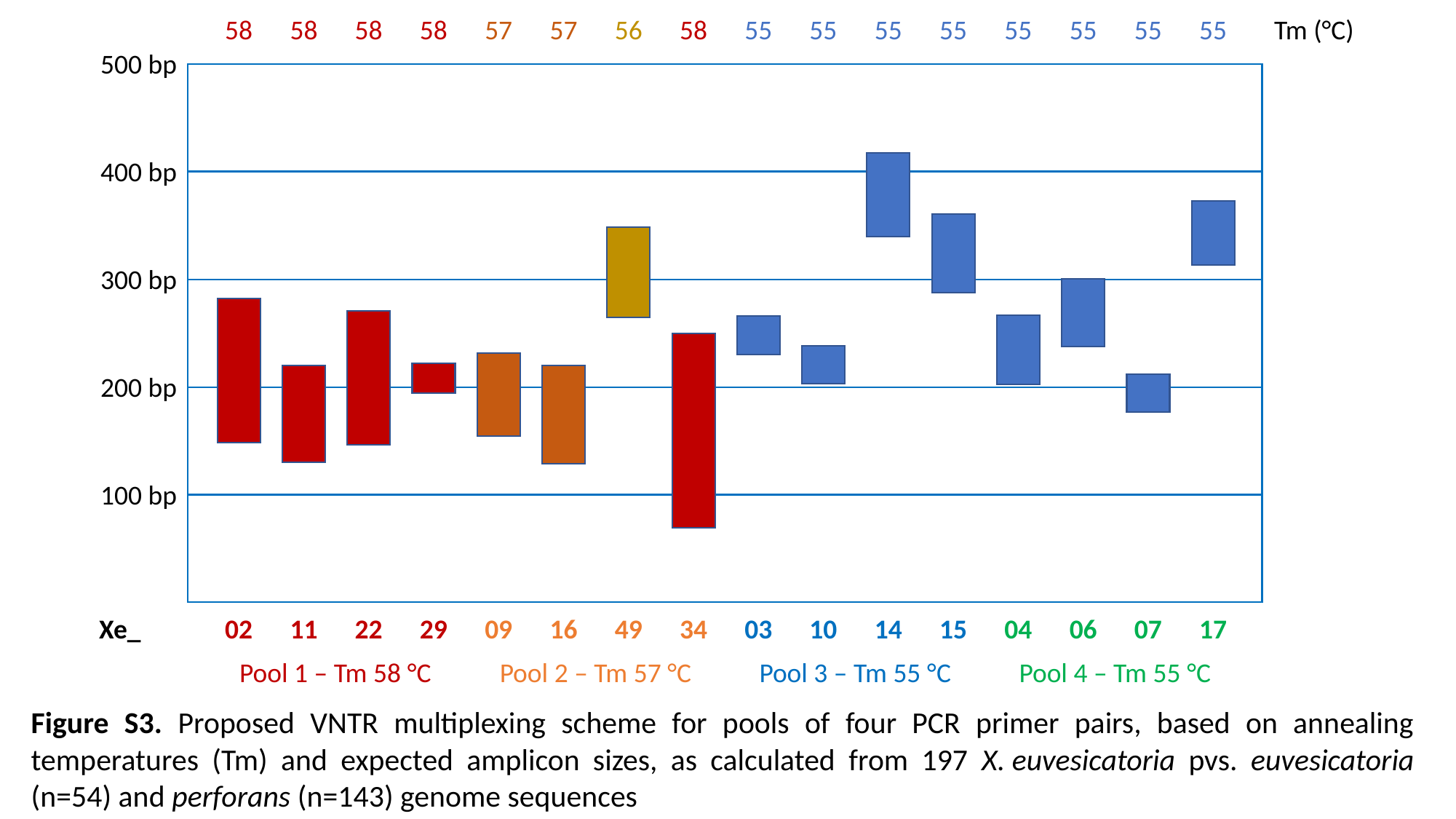

58
58
11
58
22
58
29
57
09
57
16
56
49
58
34
55
03
55
10
55
14
55
15
55
04
55
06
55
07
55
17
Tm (°C)
500 bp
400 bp
300 bp
200 bp
100 bp
Xe_
02
Pool 1 – Tm 58 °C
Pool 2 – Tm 57 °C
Pool 3 – Tm 55 °C
Pool 4 – Tm 55 °C
Figure S3. Proposed VNTR multiplexing scheme for pools of four PCR primer pairs, based on annealing temperatures (Tm) and expected amplicon sizes, as calculated from 197 X. euvesicatoria pvs. euvesicatoria (n=54) and perforans (n=143) genome sequences
