# Supplementary material for: Molecular Epidemiology of Xanthomonas euvesicatoria Strains from the Balkan Peninsula Revealed by a New Multiple-Locus Variable-Number Tandem-Repeat Analysis Scheme
Source: Microorganisms. 2021 Mar 5;9(3):536. doi: 10.3390/microorganisms9030536 (PMC8002079; doi:10.3390/microorganisms9030536)
Supplement: Supplementary file 1 [file microorganisms-09-00536-s001.zip › VANCHEVA-Table_S2.docx]

**Table S2.** Oligonucleotide primers used in this study.

| **VNTR locus** | **Oligonucleotide primer pair (5’-3’) ^1^** | **Annealing temperature (°C)** |
| --- | --- | --- |
| Xe_02 | *CGAAGCGGCTGCAGTTGTC/ TCGCGGAAGGTCATGGGCTG | 58 |
| Xe_03 | GTTGCGCTGAGCCGACTG/ *GAGCCATCACCACGCAGG | 55 |
| Xe_04 | *GGCAATCTGCAGCATCGG/ GGCATTCACCGGGCAACC | 55 |
| Xe_06 | *TCAATTCGCGTCGCGCTG/ GCCCGGATAAGCACCGCA | 55 |
| Xe_07 | *GACCCGGGAAATGCCGAA/ GCATCGGACATGCGGGAA | 55 |
| Xe_09 | AACCGGCCAACCGCTTTTAC/ *CCTGTTCGAGCAACAGGCAG | 57 |
| Xe_10 | CTTTCGCGACCGAAGCGG/ *GGCCTGGACAAAAGCGCC | 55 |
| Xe_11 | AAGTTTCGCATGGTGGGGCC/ *CTTCATGCGAGGTACCGCCC | 58 |
| Xe_14 | CGAACGGGGATCTGAGTTGC/ *CGAAACTGCAAACCGAGCAAAC | 55 |
| Xe_15 | *ATGGCATCGCGCATCGTC/ ATCGGCAGCGACGTCTGC | 55 |
| Xe_16 | CATGGCTCATCGAGCAGCAC/ *GGTCACCAGCAGGACAAGGG | 57 |
| Xe_17 | GATGGCGCGCAGCAGATC/ *TCGGGGATGGAGGCAAGC | 55 |
| Xe_22 | *CGGCAGCACCTTGCTGACC/ CAGCAGCGCGATCTGATGG | 58 |
| Xe_29 | GTGGTGGTGCGGACCGAG/ *CACCGATGCGTTCTGGCC | 58 |
| Xe_34 | CCCTCACCCGTCATCCCAAC/ *GCCTACGAATTCCGCATCGC | 58 |
| Xe_49 | *TCCAGGCCACATCGTCCG/ CGCCCAAACTCGCTACGC | 56 |

^1^ Oligonucleotide primers used for DNA sequencing are indicated by a *.
