# Supplementary material for: Molecular Epidemiology of Xanthomonas euvesicatoria Strains from the Balkan Peninsula Revealed by a New Multiple-Locus Variable-Number Tandem-Repeat Analysis Scheme
Source: Microorganisms. 2021 Mar 5;9(3):536. doi: 10.3390/microorganisms9030536 (PMC8002079; doi:10.3390/microorganisms9030536)
Supplement: Supplementary file 1 [file microorganisms-09-00536-s001.zip › VANCHEVA-Table_S3.docx]

**Table S3.** Genetic diversity parameters estimated for *X. euvesicatoria* strains from different regions of Bulgaria (B) and North Macedonia (M). Numbers are estimated from the VNTR-rep dataset.

| **Region ^1^** | **N ^2^** | **Polymorphic loci ^3^** | **eMLG ^4^** | **Simpson index D ^5^** | **H_E_ ^6^** | **A ^7^** | **Ap ^8^** |
| --- | --- | --- | --- | --- | --- | --- | --- |
| B1 | 8 | 8 | 5 | 0.857 | 0.301 | 2 | 0.5 |
| B2 | 10 | 15 | 7 | 0.933 | 0.608 | 2.68 | 0.17 |
| B3 | 17 | 16 | 4.46 | 0.654 | 0.306 | 2.39 | 0.15 |
| B4 | 10 | 14 | 6 | 0.778 | 0.286 | 2.22 | 0.02 |
| M1 - SE | 16 | 15 | 7.21 | 0.917 | 0.389 | 2.61 | 0.05 |
| M2 - E-NE | 8 | 9 | 5 | 0.857 | 0.270 | 1.63 | 0 |
| M3 - W | 19 | 9 | 4.42 | 0.608 | 0.132 | 1.68 | 0.06 |

^1^ Strains are grouped according to the region from where they originate, with four Bulgarian regions (B1 to B4) and three North Macedonian regions (SE, South East; E-NE, East-North East; W, West).

^2^ Sample size (number of strains).

^3^ Number of polymorphic loci.

^4^ eMLG, expected number of MLVA genotypes estimated from a rarefaction procedure (n=8).

^5^ Simpson index haplotypic diversity.

^6^ H_E_, Nei’s index of gene diversity.

^7^ A, allelic richness estimated from a rarefaction procedure (n=8).

^8^ Ap, private allelic richness estimated from a rarefaction procedure (n=8).
